# Comparison of whole blood cytokine immunoassays for rapid, functional immune phenotyping in critically ill patients with sepsis

**DOI:** 10.1186/s40635-023-00556-w

**Published:** 2023-10-13

**Authors:** Anthony S. Bonavia, Abigail Samuelsen, Menglu Liang, Jodi Hanson, Daniel McKeone, Zissis C. Chroneos, E. Scott Halstead

**Affiliations:** 1 Department of Anesthesiology and Perioperative Medicine, Penn State Milton S. Hershey Medical Center, Hershey, PA USA; 2 Department of Epidemiology and Biostatistics, School of Public Health, University of Maryland, Baltimore, MD USA; 3 Cellular Technology, Shaker Heights, OH USA; 4 Department of Pediatrics, Penn State Milton S. Hershey Medical Center, Hershey, PA USA

**Keywords:** Sepsis, Immunoassay, Cytokines, Critical Illness, Humans

## Abstract

**Background:**

Sepsis is characterized by highly heterogeneous immune responses associated with a spectrum of disease severity. Methods that rapidly and sensitively profile these immune responses can potentially personalize immune-adjuvant therapies for sepsis. We hypothesized that the ELLA microfluidic approach to measure cytokine production from the whole blood of septic and critically ill patients would deliver faster, more precise results than the existing optic-driven ELISpot quantification. We tested our hypothesis by measuring ex vivo*-*stimulated production of TNF and IFNγ in critically ill and septic patients (*n* = 22), critically ill and non-septic patients (*n* = 10), and healthy volunteers (*n* = 10) through both ELLA and ELISpot immunoassays. Blood samples were subjected to one of three stimulants for 4 h or 18 h durations during days 1, 7–10, and 14 of critical illness. Stimulants for lymphocytes included anti-CD3/anti-CD28 and phorbol 12-myristate 13-acetate (PMA), whereas LPS was used for monocytes. Stimulated TNF and IFNγ concentrations were then associated with 30-day mortality.

**Results:**

Both ELISpot and ELLA immunoassays showed substantial agreement in TNF concentrations post 4 h and 18 h LPS stimulation, with concordance correlation coefficients at 0.62 and 0.60, respectively. ELLA had a broad dynamic measurement range and provided accurate TNF and IFNγ readings at both minimal and elevated cytokine concentrations (with mean coefficients of variation between triplicate readings at 2.1 ± 1.4% and 4.9 ± 7.2%, respectively). However, there was no association between the ELLA-determined cytokine concentrations on the first day of critical illness and 30-day mortality rate. In contrast, using the ELISpot for cytokine quantification revealed that non-survivors had reduced baseline TNF levels at 18 h, decreased LPS-induced TNF levels at 18 h, and diminished TNF levels post 4 h/18 h anti-CD3/28 stimulation.

**Conclusions:**

Our study affirms the feasibility of obtaining dependable immune phenotyping data within 6 h of blood collection from critically ill patients, both septic and non-septic, using the ELLA immunoassay. Both ELLA and ELISpot can offer valuable insights into prognosis, therapeutic strategies, and the underlying mechanisms of sepsis development.

**Supplementary Information:**

The online version contains supplementary material available at 10.1186/s40635-023-00556-w.

## Background

Sepsis is defined as the life-threatening organ dysfunction that is caused by a dysregulated host response to infection [1]. The World Health Organization considers it an epidemic, as it accounts for over 11 million global deaths annually [2]. Secondary bacterial and fungal infections develop in up to 40% of patients in the post-sepsis period and dramatically increase mortality rates [3–5]. Sepsis-induced “immune paralysis” is believed to be the cause underlying this high rate of secondary infections. While immune paralysis has historically been defined as the impaired ability to produce TNF in response to endotoxin exposure [6–8], increasing awareness of the severity of lymphocyte apoptosis and adaptive immune dysfunction in sepsis has created a need to concurrently interrogate lymphocyte function. In the context of highly heterogeneous immune responses in septic patients, however, a rapid method of quantifying the severity of immune paralysis direly needed. This may allow clinicians to intervene early in the disease process by administering immune adjuvant therapy to bolster a paralyzed immune system, thereby preventing secondary infections [9, 10].

Towards this goal, a recent study demonstrated that diminished production of both tumor necrosis factor (TNF) and interferon (IFN)γ following ex vivo stimulation of whole blood from septic patients distinguished between sepsis survivors and non-survivors [11]. This investigation used an enzyme-linked immunospot (ELISpot) assay to measure cytokine concentrations*,* and represents a pivotal advance towards developing a rapid, clinically useful, and bedside-compatible test for immune paralysis in sepsis. The ELISpot immunoassay offers single-cell resolution and cytokine quantification on a per-cell basis. However, it is also susceptible to imaging artifact and requires approximately 24 h to yield results [11]. These drawbacks are not trivial in a rapidly evolving and potentially fatal disease.

We thus hypothesized that equivalent, accurate and precise results could be obtained more rapidly using an automated, microfluidic-based immunoassay rather than an image-based one. Our hypothesis was based on our recent study comparing ELLA and ELISpot-based immune interrogation in a cohort of healthy volunteers [12]. Like the study by Mazer et al., the ELLA immunoassay could be performed with whole blood, precluding the need to isolate PBMC [11, 12]. However, it offers the additional advantages of a 90-min turn-around time, full automation, and cytokine detection spanning a wide dynamic measurement range. Disadvantages of ELLA include cost and limited clinical validation.

In the present study, we compared the performance of ELLA and ELISpot assays in critically ill septic and non-septic patients and healthy volunteers. Our primary objectives were technical, focusing on (1) the concordance between ELISpot and ELLA-measured TNF and IFNγ concentrations, (2) variation between replicate measurements (precision), and (3) whether cytokine concentrations following 4 h of ex vivo stimulation recapitulated results at 18 h. The secondary objective was to investigate whether stimulated cytokine production measured over the first 2 weeks of critical illness predicted 30-day survival.

## Methods

### Study design and patient enrollment

This prospective, observational trial was performed at a quarternary care, academic medical center and included critically ill, adult patients and healthy control volunteers recruited between 11/2021 and 10/2022. All participants were enrolled at the Penn State Milton S Hershey Medical Center, following study approval by the Human Study Protection Office (IRB #15328 and #10357) and after obtaining informed consent from each patient or from their legally authorized healthcare representative. A Modified Early Warning Scoring (MEWS)-based algorithm [13, 14] was used to query the electronic medical record for patients developing clinical deterioration, and their potential recruitment into critically ill and septic or critically ill and non-septic (CINS) cohorts. To avoid selection bias, at least two study investigators independently reviewed the medical records of all patients identified by electronic screening and evaluated them for further study inclusion, according to the criteria listed below. Agreement between both investigators was required prior to study recruitment.

### Inclusion criteria

Consecutive septic patients who met enrollment criteria of > 18 years and < 48 h of critical illness were eligible for enrollment in the sepsis cohort. Sepsis was defined by a change in sequential organ failure assessment (SOFA) score of two or more, in the setting of clinically suspected or microbiologically proven infection [1]. Critical illness was defined by the need for continuous intravenous infusion of vasopressors to maintain a mean arterial pressure of ≥ 65 mmHg, and/or the need for continuous respiratory support and monitoring. CINS patients fulfilled criteria for critical illness but not sepsis. Non-survivors were defined as critically ill patients who died $\le$ 30 days following study enrollment. Healthy volunteers included non-hospitalized adults who did not have major medical comorbidities, did not take immunosuppressive medications, and did not have suspected or diagnosed immune disorders.

### Exclusion criteria

Patients having active hematologic malignancies (leukemia or lymphoma) and those who were on immunomodulating therapies were excluded. We also excluded (1) patients receiving chronic steroid therapy outside the setting of acute sepsis, (2) patients with HIV infection with a CD4 count < 200 cells/mm^3,^ (3) recipients of solid organ transplantation who were on long-term immunotherapy, (4) patients who received chemotherapy or radiotherapy within the prior 30 days, and (5) patients with systemic autoimmune diseases, such as systemic lupus erythematosus or rheumatoid arthritis. Patients who were given corticosteroids as part of their sepsis management were not excluded from the study.

### Clinical variables

Clinical data for septic and CINS patients were obtained from the electronic medical record, while the patient was hospitalized, and via 30-day phone interview for patients who were successfully discharged from the hospital. To quantify illness severity, we utilized the Charlson Comorbidity Index as an aggregate measure of the burden of chronic medical comorbidities [15], and the Acute Physiology and Chronic Health Evaluation (APACHE II) and Sequential Organ Failure Assessment (SOFA) scores to quantify severity of acute illness [16–18].

### Processing of blood samples

All venous blood samples were collected in tubes containing sodium heparin within 48 h of the onset of critical illness ± sepsis (study day 1), on study days 7–10 and on study day 14, for survivors who remained hospitalized during that period. Samples were kept at on ice until time of processing. Leukocyte count and differential cell profile was assessed from whole blood collected in a tube containing ethylenediamine tetraacetic acid (EDTA). Failed assays could not be repeated, due to need for fresh blood samples for immune assays and sample processing/testing times ranging from of 6 h (ELLA assay with 4 h incubation time) to 24 h (ELISpot assay with 18 h incubation time).

### ELISpot analysis

Whole blood samples from each study participant were exposed to one of three stimulants (LPS, PMA, anti-CD3/anti-CD28), or remained unstimulated. Stimulation was further performed for either 4 h or 18 h, resulting in a total of eight treatment conditions per participant. Assays were performed in duplicate resulting in a total of 672 assays, each measuring TNF and IFNγ concentrations, as described below.

Double-color, enzymatic-based ELISpot plates allowed the simultaneous measurement of both TNF and IFNγ in blood. Our selection of dual-color, enzymatic measurement strips was informed by the need for parallel comparison of ELISpot with the ELLA microfluidic-based, cytokine measurement system. ELISpot polyvinylidene difluoride strip plates were activated with ethanol, rinsed, and then incubated overnight with capture antibodies, per manufacturer’s instructions (ImmunoSpot, Cellular Technology, Cleveland, OH). 50 $\upmu$l of whole blood was then diluted tenfold in complete cell culture media supplemented with 1% glutamine, and added to ELISpot wells that contained one of three stimulants: (1) 500 ng/ml of anti-CD3 (Cat# 300302, Biolegend, San Diego, CA) with 2.5 $\upmu$g/ml anti-CD28 (Cat# 302902, Biolegend), (2) 0.08 $\upmu$M (49.3 ng/ml) phorbol 12-myristate 13-acetate (PMA) with 1.3 $\upmu$M (0.97 $\upmu$g/ml) ionomycin (Thermo Fisher Scientific, Waltham, MA), or (3) 2.5 ng/ml LPS from *Salmonella enterica* strain abortus equi (Cat# L1887, Sigma-Aldrich, St. Louis, MO). Concentrations used were based on previously published studies [11, 12]. All steps were undertaken using aseptic technique, in a laminar flow hood, to reduce the likelihood of endotoxin contamination. Duplicate samples were incubated for either 4 or 18 h at 5% CO_2_ and 37 °C. The strip plates were washed and anti-human TNF and anti-human IFNγ detection antibodies, conjugated to biotin and fluorescein isothiocyanate (FITC), respectively, were then added to each well. After 2 h, the strip plates were again washed and tertiary solutions of streptavidin alkaline phosphatase and anti-FITC horseradish peroxidase were added, followed by another wash and the addition of blue (TNF spots) and red (IFNγ spots) developer solutions.

A cellular technology series 6 Immunospot Universal Analyzer, running ImmunoSpot 7.0 (Cellular Technology Analyzers, Shaker Heights, OH), was used to scan wells for spot count. Spot detection parameters were optimized following blinded, aggregate review of all ELISpot images included in the study. Once the detection parameters were optimized for analysis, spot counts were determined in a fully automated fashion. Quality control was subsequently performed, with investigator blinded to study cohort, to ensure that image artifacts were not inadvertently counted as spots by the image analyzer.

### ELLA microfluidic immunoassay

50 $\upmu$L of whole blood was added to 450 μL of HEPES-buffered RPMI media, in a 1.6 ml polypropylene tube containing one of three stimulants: (1) anti-CD3 with anti-CD28, (2) 16 nM PMA with 1.3 $\upmu$M ionomycin, or (3) 500 pg/ml LPS from *Salmonella enterica* strain abortus equi. Concentrations were based on previously published studies [8, 11]. Blood was incubated at 37 °C for either 4 h or 18 h together with unstimulated blood (4 h in cell culture media), resulting in a total of seven samples per patient and a total of 294 ELLA assays. Following incubation, samples were centrifuged at 1000 × g for 5 min. Supernatants from ex vivo blood stimulation were analyzed for IFNγ and TNF by Simple Plex analysis (human SPCKA-PS cartridges including IFNγ 3rd generation, and TNF-a 2nd generation) using the ELLA microfluidic immunoassay system, per manufacturer’s instructions (ProteinSimple, San Jose, CA). The ELLA system measured and reported triplicate values for TNF and for IFNγ concentrations, from each of these 294 assays. Sample results were reported using Simple Plex Runner v.3.7.2.0 (ProteinSimple) and were available within 90 min.

### Statistics

A sample size of 22 septic patients was selected based on a recently published and comparable investigation, assessing ELISpot analysis for whole blood functional immunotyping in sepsis [11]. Statistical analysis was performed by Prism v10.0.2 (GraphPad Software, San Diego, CA) and with R statistical software (v4.2.2, R Core Team 2022). Statistical tests were two-tailed with a level of significance set at *α* = 0.05. Descriptive statistics were used to characterize the study population, with continuous variables reported as mean ± standard deviation and categorical variables reported as counts with proportions. Given the skewed distributions of cytokine concentrations and cell counts, these values were log-transformed (using base 2) before statistical analyses were conducted. To represent all measured values and to enable the statistical comparison of log_2_-transformed values between groups, cytokine concentrations with null values were assigned a value corresponding to the theoretical lower limit of detection (10^–3^). ELISpot measurements that were above the limit of detection were set to the highest spot count that could be reliably measured for that cytokine.

Differences between cohorts were assessed using Analysis of Variance (ANOVA) or the student’s *t* test for continuous variables, and the Chi-square test or Fisher’s exact test for categorical variables. Concordance between concentrations of the same cytokine with different lengths of stimulation (4 h versus 18 h) was assessed by calculating Lin’s concordance correlation [19]. Spearman’s rank correlation coefficient was used to compare cytokine concentrations in the same blood sample, measured by ELLA versus ELISpot assays.

## Results

The demographic profiles and clinical outcomes for 32 critically ill patients (22 with sepsis and 10 non-septic controls) are shown in Table 1. For culture-proven infections, the microbial sources of sepsis are enumerated in Table 2. The mean age of the healthy volunteers was 39 years (range 26–58 years) and 50% were male; one was Asian, and nine were Caucasian.

**Table 1:** Patient demographics

	Critically ill, septic (*n* = 22)	Critically ill, non-septic (*n* = 10)
Age, mean (range)	58 (31–91)	72 (59–88)
*Sex*		
Female	15 (68.2%)	5 (50%)
*Race*		
African American	0	2 (20%)
White	20 (90.0%)	8 (80%)
Asian	1 (4.5%)	0
Hispanic	1 (4.5%)	0
*Medical comorbidities*		
Cancer	8 (36.4%)	3 (30%)
Cardiovascular disease	12 (54.5%)	7 (70%)
Peripheral vascular disease	3 (13.6%)	3 (30%)
Diabetes	9 (40.9%)	3 (30%)
Gastrointestinal disease	7 (31.8%)	4 (40%)
Hepatic disease	1 (4.5%)	1 (10%)
Hyperlipidemia	9 (40.9%)	5 (50%)
Hypertension	12 (54.5%)	6 (60%)
Kidney or urologic disease	8 (36.4%)	3 (30%)
Cerebrovascular or neurologic disease	8 (36.4%)	3 (30%)
Obesity	6 (27.3%)	5 (50%)
Respiratory disease	7 (31.8%)	5 (50%)
Substance abuse	0	1 (10%)
Thyroid disease	5 (22.7%)	3 (30%)
*Severity of illness*		
APACHE II score	23 ± 6.2	25.3 ± 4.7
SOFA score	8 ± 3.5	8 ± 3.1
Charlson Comorbidity Index	6 ± 2.9	6.4 ± 2.4
Patients receiving stress-dosed hydrocortisone (*n*)	4 (18%)	0
Mean daily hydrocortisone dose amongst recipients (mg)	137 ± 36	-
Mean duration of hydrocortisone amongst recipients (d)*	5.3 ± 3.0	–
*Laboratory values*		
Leukocyte count (× 10^3^/µl)	21.184 ± 10.5	13.797 ± 9.8
Absolute lymphocyte count (× 10^3^/µl)	0.889 ± .63	1.845 ± 1.9
Absolute monocyte count (× 10^3^/µl)	0.938 ± .71	1.528 ± .70
Lactic acid (mg/dL) on admission	4.000 ± 3.5	2.528 ± 1.4
Shock (lactate > 2) on admission (*n*)	5 (22.7%)	0
*30-Day outcomes*		
Secondary infections (*n*, %)	4 (18.2%)	1 (10%)
Secondary infection with gram negative (*n*, %)	2 (9.1%)	0
In-hospital mortality rate (*n*, %)	7 (31.8%)	1 (4.5%)
30-Day mortality rate (*n*, %)	7 (31.8%)	1 (4.5%)
Mean hospital length of stay (days)	11.8 ± 6.4	14.3 ± 5.3
Mean ECOG/Zubrod score at hospital discharge	3 ± 1.9	3.1 ± 1.4
Mean ECOG/Zubrod Score at 30 Days	2.7 ± 1.3	2 ± 1.6
Hospital readmission rate (*n*, %)	2 (9.1%)	1 (4.5%)
Mean length of antibiotic therapy (days)	11.1 ± 6.9	4.3 ± 4.8
*Long-term outcomes*		
Death-free days until follow-up (days)	112 ± 98.2	173.3 ± 89.2
Discharged to nursing facility or long-term acute care hospital (*n*, %)	7 (32%)	5 (50%)

^*^Within 14 days following enrollment

**Table 2:** Microbial sources of sepsis in critically ill and septic cohort

Septic patient #	Clinical diagnosis of sepsis (negative microbial cultures)	Organism(s)	Source			
			Blood	Sputum/respiratory	Abdomen	Urine
1		*Gemella morbillorum*	X			
2		*Staphylococcus epidermidis*	X			
3	X					
4		*Escherichia coli, Aerococcus urinae*				X
5		*COVID-19*		X		
6		*Escherichia coli*	X			
7		*Escherichia coli*			X	
8		*Klebsiella pneumoniae*			X	
9		*Escherichia coli), Enterococcus faecium*	X		X	
10		*Escherichia coli*	X			X
11	X					
12		*Klebsiella pneumoniae*	X			
13	X					
14		*Candida glabrata, Candida lusitaniae*			X	
15		Anaerobic Gram-positive rods (unspeciated)			X	
16		*Proteus mirabilis*	X			
17		*Pseudomonas aerugiosa*		X		
18		*Clostridium clostridiforme*			X	
19		*Fusobacterium nucleatum*	X			
20		*Klebsiella pneumoniae, Proteus mirabilis*				X
21		*Escherichia coli*				X
22		*Group B streptococcus, Staphylococcus aureus and Stapylococcus lugdenensis*	X			

### ELISpot to measure functional immune status

**Fig. 1:**
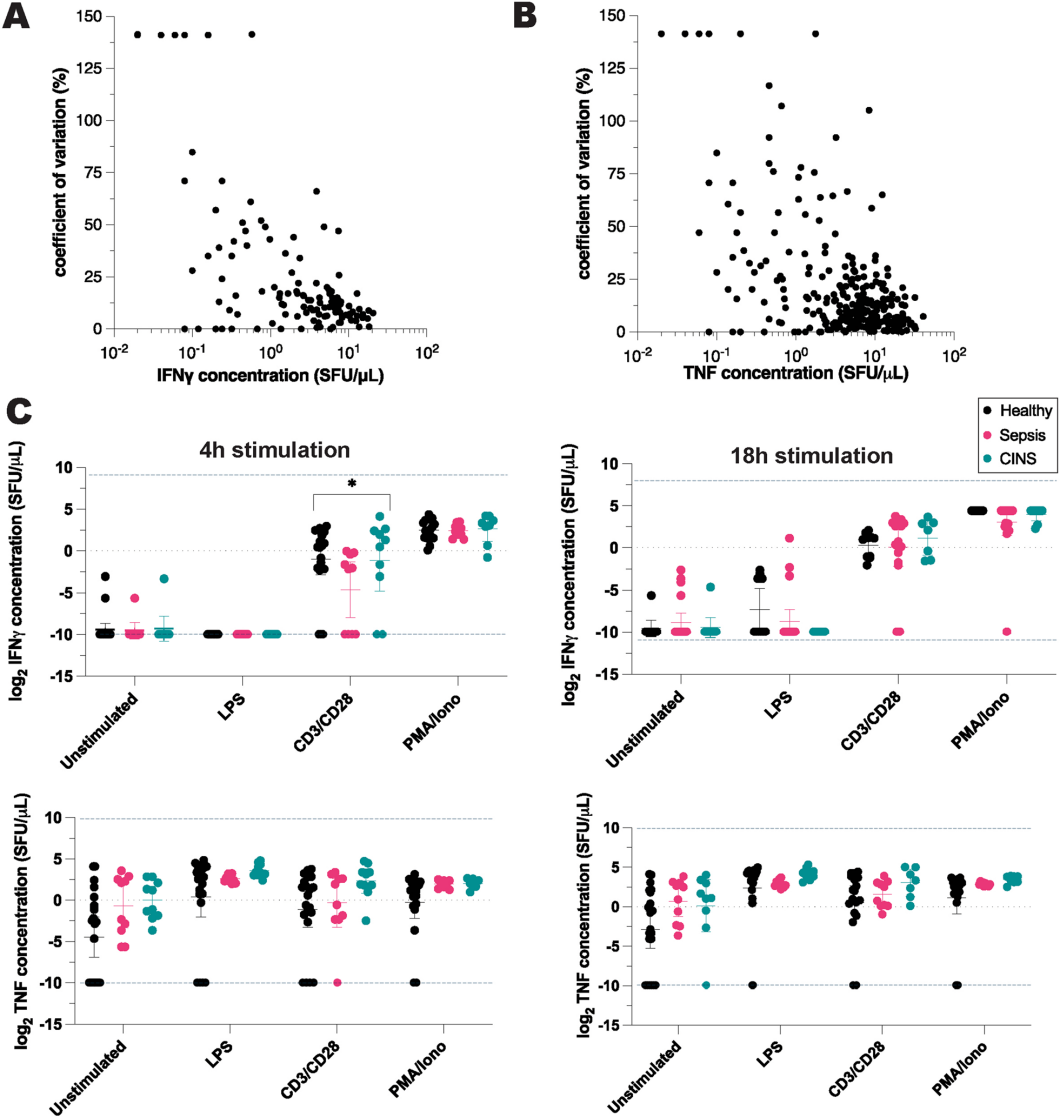
IFNγ and TNF concentrations measured by ELISpot immunoassay, following ex vivo exposure of whole blood to immune stimulants. **A** Coefficient of variation of pooled IFNγ measurements, **B** coefficient of variation of pooled TNF measurements, **C** IFNγ and TNF responses measured by ELISpot immunoassay following 4 or 18 h of exposure to immune stimulants. *CINS*  critically ill and not septic patients, *SFU*  spot forming units, *LPS*  lipopolysaccharide, *CD3/CD28*  anti-CD3 and anti-CD28 antibodies, *PMA/iono*  phorbol 12-myristate 13-acetate and ionomycin. Blue dotted lines represent upper and lower limits of detection. *P* values based on ANOVA analysis using log_2_-transformed cytokine concentrations from three patient cohorts (healthy, sepsis, CINS). *P ≤ 0.05

The ELISpot assay provides an intuitive, visual representation of ex vivo immune-reactivity, where each blue spot represents one TNF-producing cell (or spot-forming unit, SFU) and each red spot represents one IFNγ-producing cell (Additional file 1: Fig. S1). Of the 16 ELISpot wells (4 stimulation conditions, two timepoints, and performed in duplicate) analyzed from each of 42 experimental subjects, one septic patient’s ELISpot assay failed, resulting in a total of 656 ELISpot assays each yielding a single TNF and IFNγ measurement. 56 (4.3%) of these measurements were above the limit of detection, and 66 (5%) could not be quantified due to significant optical artifact noted in the quality control step, necessitating sample exclusion. Of 1190 quantifiable cytokine measurements (SFUs), 328 (100%) were the result of unstimulated blood, 323 (98.5%) resulted from blood exposed to LPS, 234 (71.3%) resulted from blood exposed to PMA/ionomycin, and 305 (93%) resulted from blood exposed to anti-CD3/anti-CD28. While 4 h of PMA/ionomycin stimulation produced spot counts that were within the quantifiable range, 34% of PMA/ionomycin samples exposed to prolonged (18 h) stimulation demonstrated intense cytokine production (confluent spots) that were above the IFNγ or TNF limits of detection. The mean coefficient of variation between SFUs measured from duplicate samples was 26 ± 37% (range 0–141%) for IFNγ measurements (Fig. 1A), and 22 ± 29% (range 0–141%) for TNF measurements (Fig. 1B).

### ELLA microfluidic-based, simple plex immunoassay to measure functional immune status

**Fig. 2:**
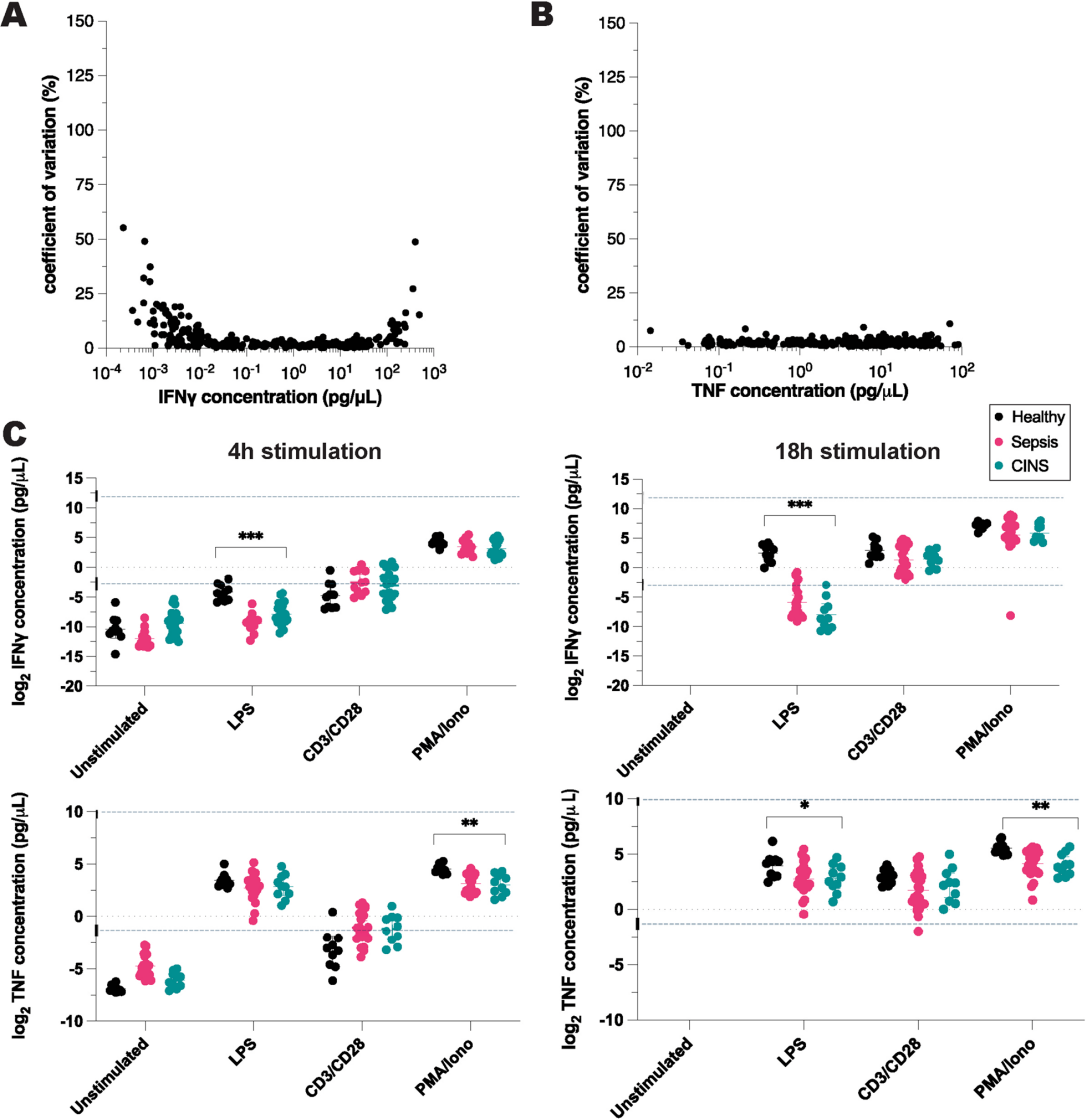
IFNγ and TNF concentrations measured by ELLA immunoassay, following ex vivo exposure of whole blood to immune stimulants. **A** Coefficient of variation of pooled IFNγ measurements, **B** coefficient of variation of pooled TNF measurements, **C** IFNγ and TNF responses measured by ELISpot immunoassay following 4 or 18 h of exposure to immune stimulants. *CINS*  critically ill and not septic patients, *LPS*  lipopolysaccharide, *CD3/CD28*  anti-CD3 and anti-CD28 antibodies, *PMA/iono*  phorbol 12-myristate 13-acetate and ionomycin. Blue dotted lines represent upper and lower limits of reliable detection provided by manufacturer. *P* values based on ANOVA analysis using log_2_-transformed cytokine concentrations from three patient cohorts (healthy, sepsis, CINS). *P ≤ 0.05, **P ≤ 0.01, ***P ≤ 0.001

ELLA assay failed in 34 (1.9%) of 1764 possible measurements. Of the 1730 resulting cytokine measurements, 243 (96%) derived from unstimulated blood, 484 (96%) were the result of LPS stimulation, 502 (99.6%) were the result of PMA stimulation, and 502 (99.4%) were the result of anti-CD3/anti-CD28 stimulation. The mean coefficient of variation between triplicate measurements was 4.9 ± 7.2% (range 0–55%) for IFNγ (Fig. 2A), and 2.1 ± 1.4% (range 0–11%) for TNF (Fig. 2B).

### Inter-assay comparison

ELLA and ELISpot differ with respect to the type of information they provide. While ELLA assay reports raw cytokine concentrations, which can then be normalized to cell count (giving a concentration of pg/cell), ELISpot-measured SFU (number of cytokine-producing cells) represents the proportion of leukocytes, per blood volume, producing the cytokine of interest. Due to the absence of ‘standards’ used to generate a dose–response curve, we could not define or compare accuracy between immunoassays. However, we noted that IFNγ concentrations measured by ELLA were highly correlated with ELISpot SFU’s (Spearman r of 0.73–0.84), with lower correlation measures for TNF (Spearman r of 0.42–0.43) (Additional file 1: Fig. S2).

### Cytokine responses to whole blood stimulation, ex vivo, in each experimental cohort

Comparison of cytokine concentrations in stimulated versus unstimulated whole blood demonstrated that spontaneous cytokine production by unstimulated whole blood is low (Additional file 1: Fig. S3). This obviated the need to define immune response by an increase (‘delta’) in cytokine concentration between stimulated and unstimulated conditions. Functional immune response was, therefore, defined by post-stimulant TNF and IFNγ concentrations alone.

The exposure of whole blood to lymphocyte stimulants, namely, anti-CD3/anti-CD28 and PMA/ionomycin, consistently induced IFNγ production in both healthy volunteers and critically ill patients, regardless of septic status (Figs. 1C and 2C). Notably, when IFNγ production was triggered by LPS, significant differences were observed through ELLA-based cytokine quantification after 4 and 18 h of stimulation (Fig. 2C). Such differences were not identifiable using the ELISpot method (Fig. 1C). In a similar vein, variations in PMA-induced TNF levels across patient groups, as measured by ELLA at both 4 h and 18 h, went undetected by ELISpot (Figs. 1C and 2C). The observed variations, especially in low concentrations of LPS-induced IFNγ and high concentrations of PMA-induced TNF, underscore the expansive dynamic measurement range offered by ELLA.

**Fig. 3:**
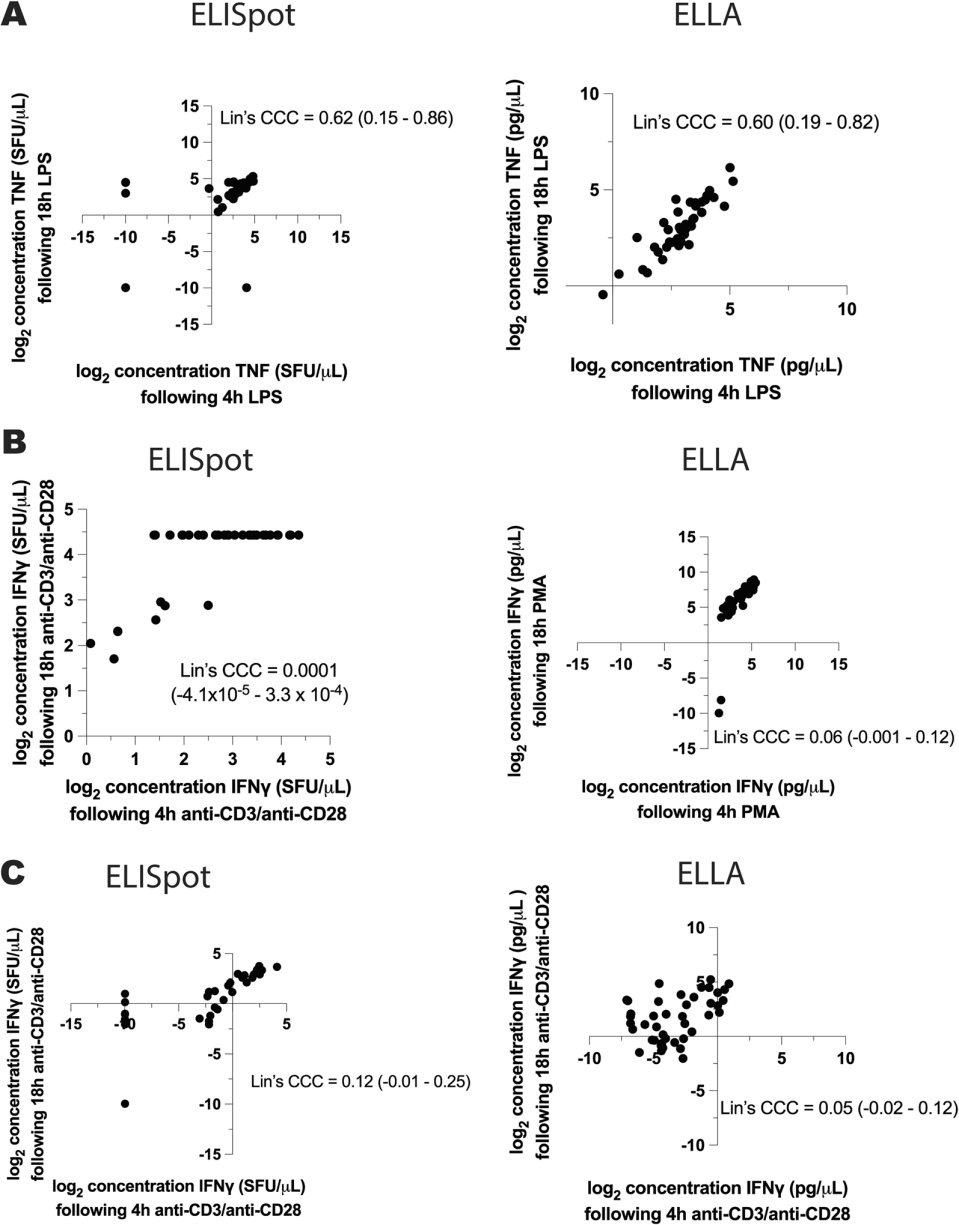
Scatterplots comparing cytokine production following 4 h and 18 h ex vivo exposure of whole blood to immune stimulants. **A** LPS-stimulated TNF production at 4 h versus 8 h measured by ELISpot versus ELLA immunoassay, **B** PMA-stimulated IFNγ production at 4 h versus 8 h measured by ELISpot versus ELLA immunoassay, **C** anti-CD3/anti-CD28-stimulated IFNγ production at 4 h versus 8 h measured by ELISpot versus ELLA immunoassay. *Lin’s CCC*  Lin’s concordance correlation coefficient, with 95% confidence interval for two-tailed test

While longer ex vivo blood stimulation predictably causes increased cytokine production in all patient cohorts, we hypothesized that critical patterns in stimulant-induced cytokine concentrations could be detected after only 4 h of whole blood stimulation (i.e., patterns of cytokine production following only 4 h of stimulation would correspond with those at 18 h). To measure the agreement between 4 and 18 h measurements, we calculated Lin’s concordance correlation coefficient (Fig. 3). The highest correlation between 4 and 18 h cytokine measurements was observed following LPS stimulation (Fig. 3A), with correlation coefficients of 0.62 and 0.60 with ELISpot and ELLA assays, respectively. There was poor correlation between cytokine concentrations at 4 h and 18 h when blood was stimulated with PMA (Fig. 3B) and anti-CD3/anti-CD28 antibodies (Fig. 3C), irrespective of the immune assay that was used.

### Stimulated IFNγ and TNF concentrations and mortality in critically ill, septic, and non-septic patients

Our secondary study objective was to analyze whether there was an association between cytokine production, measured over the first 2 weeks of critical illness, and mortality. We hypothesized that decreased IFNγ and TNF production, following exposure of whole blood to ex vivo stimulation, would predict 30-day mortality in critically ill patients. Notably, there were no significant difference between APACHE II (*p* = 0.696), SOFA (*p* = 0.338) or Charlson comorbidity (*p* = 0.563) scores and patient survival, in our cohort of critically ill patients.

**Fig. 4:**
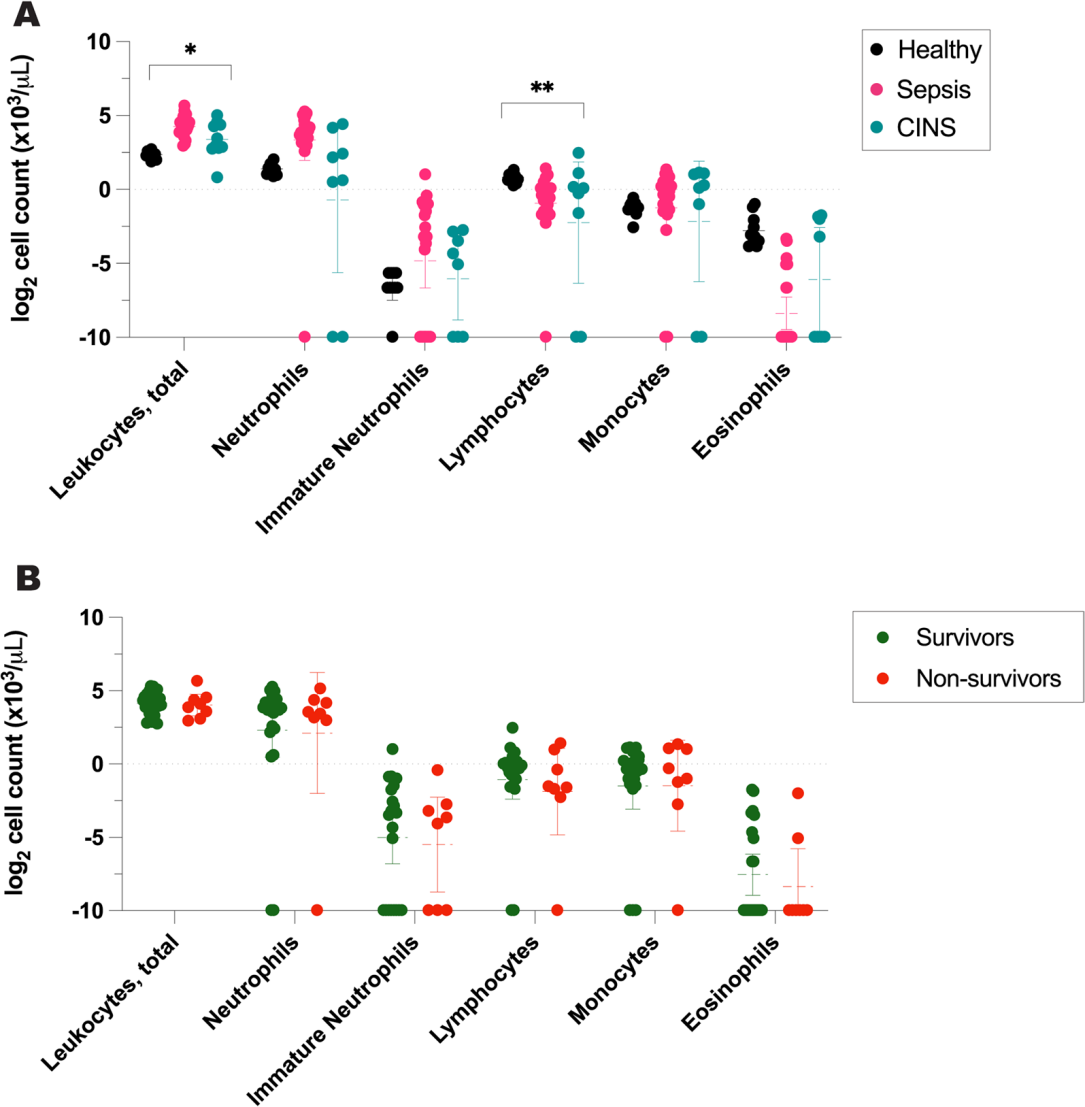
Comparison of log_2_-transformed leukocyte counts between patient cohorts. **A** Cell counts in healthy, critically ill and septic patients, and critically ill and non-septic patients with *P* values based on ANOVA, **B** cell counts in survivors versus non-survivors with *P* values based on two-tailed *t* test.* CINS* critically ill and not septic patients. *P ≤ 0.05, **P ≤ 0.01

Immune paralysis is a phenomenon defined by a deficient cellular immune response. Given the significant differences in leukocyte counts between cohorts (Fig. 4), we evaluated cytokine production both on a per-cell and on a per-volume basis. Specifically, IFNγ induced by anti-CD3/anti-CD28 and PMA was adjusted to blood lymphocyte count [11, 20], while TNF production stimulated by LPS was adjusted to blood monocyte count. Although there were significant differences in the absolute lymphocyte counts among healthy, septic, and CINS patients, absolute monocyte counts remained consistent between cohorts (Fig. 4A) [21]. Moreover, the distribution of leukocyte populations showed no variance between critically ill survivors and non-survivors (Fig. 4B).

**Fig. 5:**
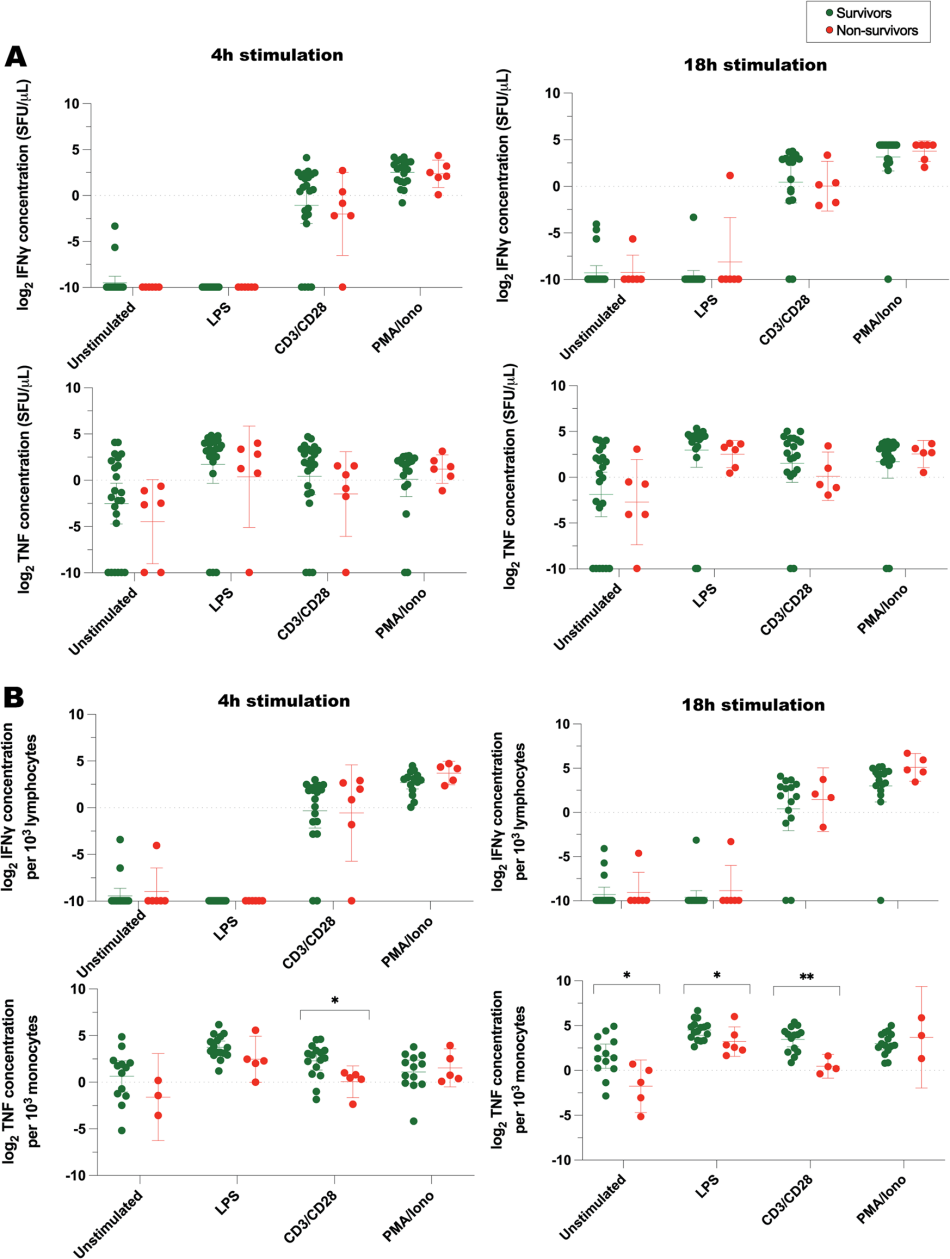
IFNγ and TNF concentrations measured by ELISpot immunoassay, following ex vivo exposure of whole blood to immune stimulants. **A** represents log_2_-transformed IFNγ and TNF concentrations, while **B** represents corresponding cytokine concentrations when normalized to number of cytokine-producing cells (lymphocytes or monocytes). Bars represent mean with 95% confidence interval. *SFU*  spot forming units, *LPS*  lipopolysaccharide, *CD3/CD28*  anti-CD3 and anti-CD28 antibodies, *PMA/Iono*  phorbol 12-myristate 13-acetate and ionomycin. *n* = 10 healthy volunteers, 24 survivors, 8 non-survivors (7 critically ill and septic and 1 critically ill and non-septic). *P ≤ 0.05, **P ≤ 0.01, based on two-tailed *t* test of log_2_-transformed cytokine concentrations from survivors versus non-survivors

**Fig. 6:**
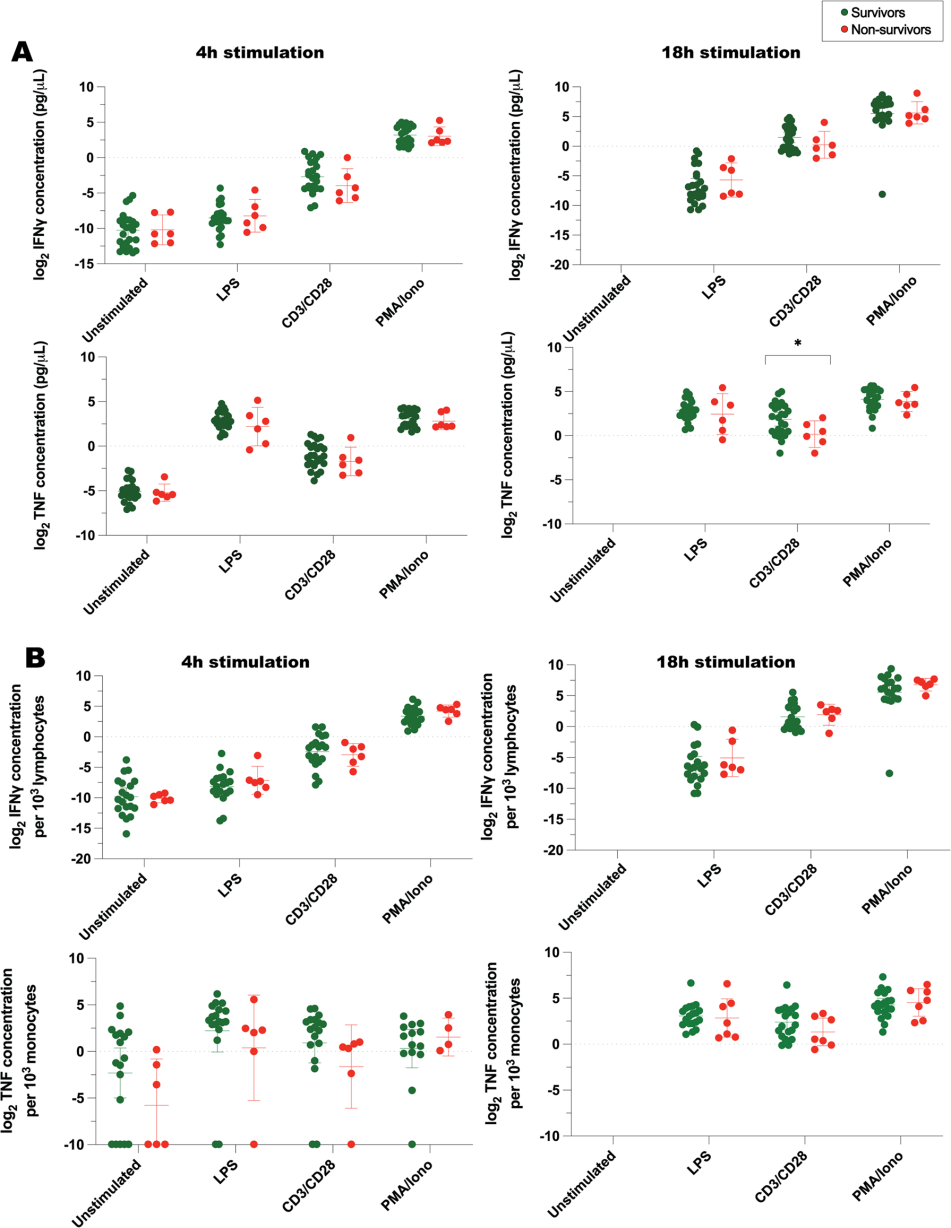
IFNγ and TNF concentrations measured by ELLA immunoassay, following ex vivo exposure of whole blood to immune stimulants. **A** represents log_2_-transformed IFNγ and TNF concentrations, while **B** represents corresponding cytokine concentrations when normalized to number of cytokine-producing cells (lymphocytes or monocytes). Bars represent mean with 95% confidence interval. *LPS* lipopolysaccharide, *CD3/CD28*  anti-CD3 and anti-CD28 antibodies, *PMA/iono*  phorbol 12-myristate 13-acetate and ionomycin. *n* = 10 healthy volunteers, 24 survivors, 8 non-survivors (7 critically ill and septic and 1 critically ill and non-septic). *P ≤ 0.05, **P ≤ 0.01, based on two-tailed *t* test of log_2_-transformed cytokine concentrations from survivors versus non-survivors

**Fig. 7:**
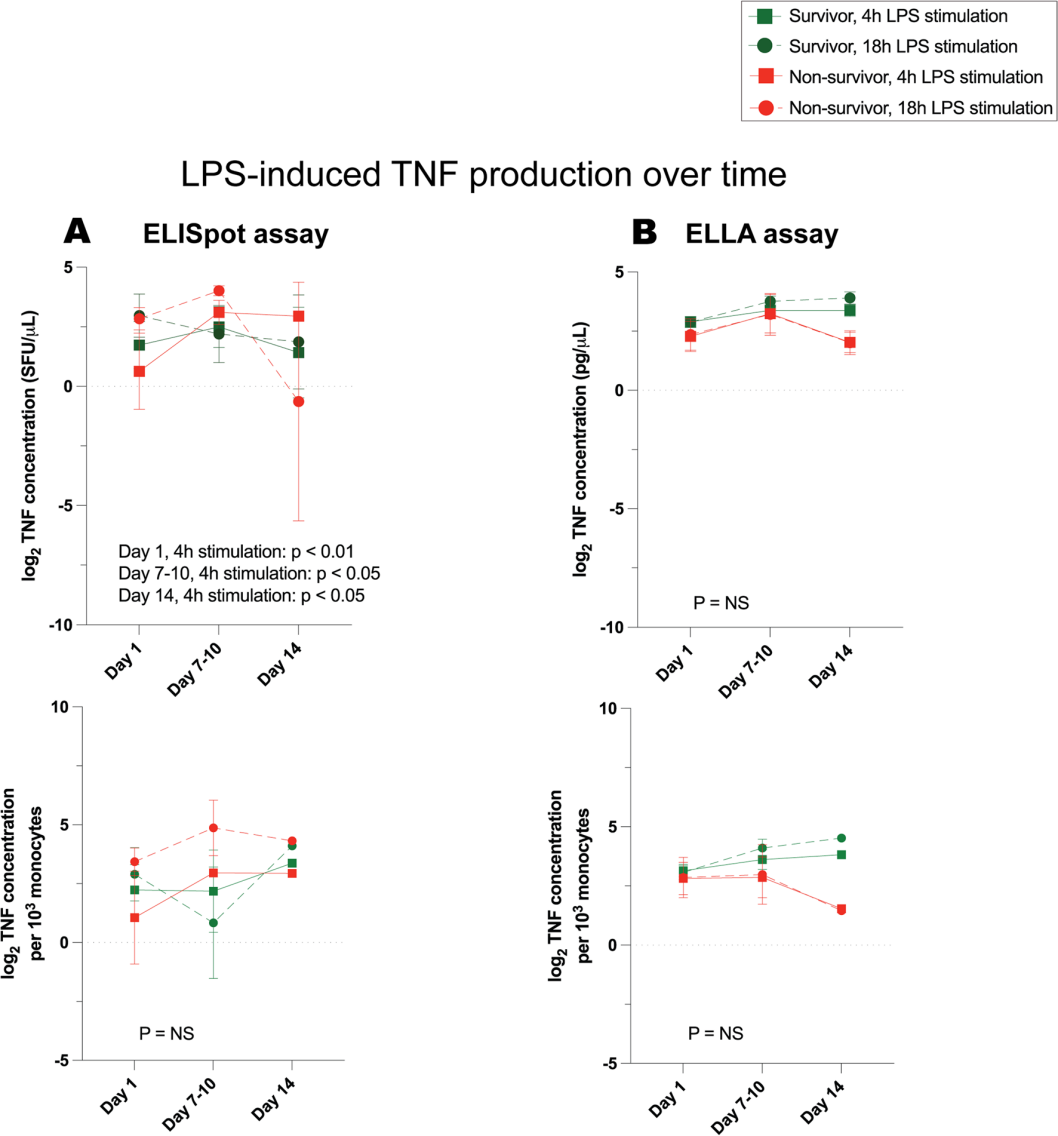
TNF production, over time, in response to LPS stimulation of whole blood from critically ill survivors versus non-survivors. **A** Log_2_-transformed TNF concentration as measured by ELISpot immunoassay, **B** log_2_-transformed TNF concentration as measured by ELLA immunoassay. Top panels illustrate cytokine production per microliter of blood, and corresponding bottom panels illustrate cytokine production per 10^3^ monocytes. Day 1 denotes the day of study enrollment; dots represent mean values ± SEM. *P* values denote significant differences between stimulated TNF production in whole blood of survivors versus non-survivors, based on linear mixed-effects analysis. At day 1, *n* = 24 survivors, 8 non-survivors (7 critically ill and septic and 1 critically ill and non-septic)

**Fig. 8:**
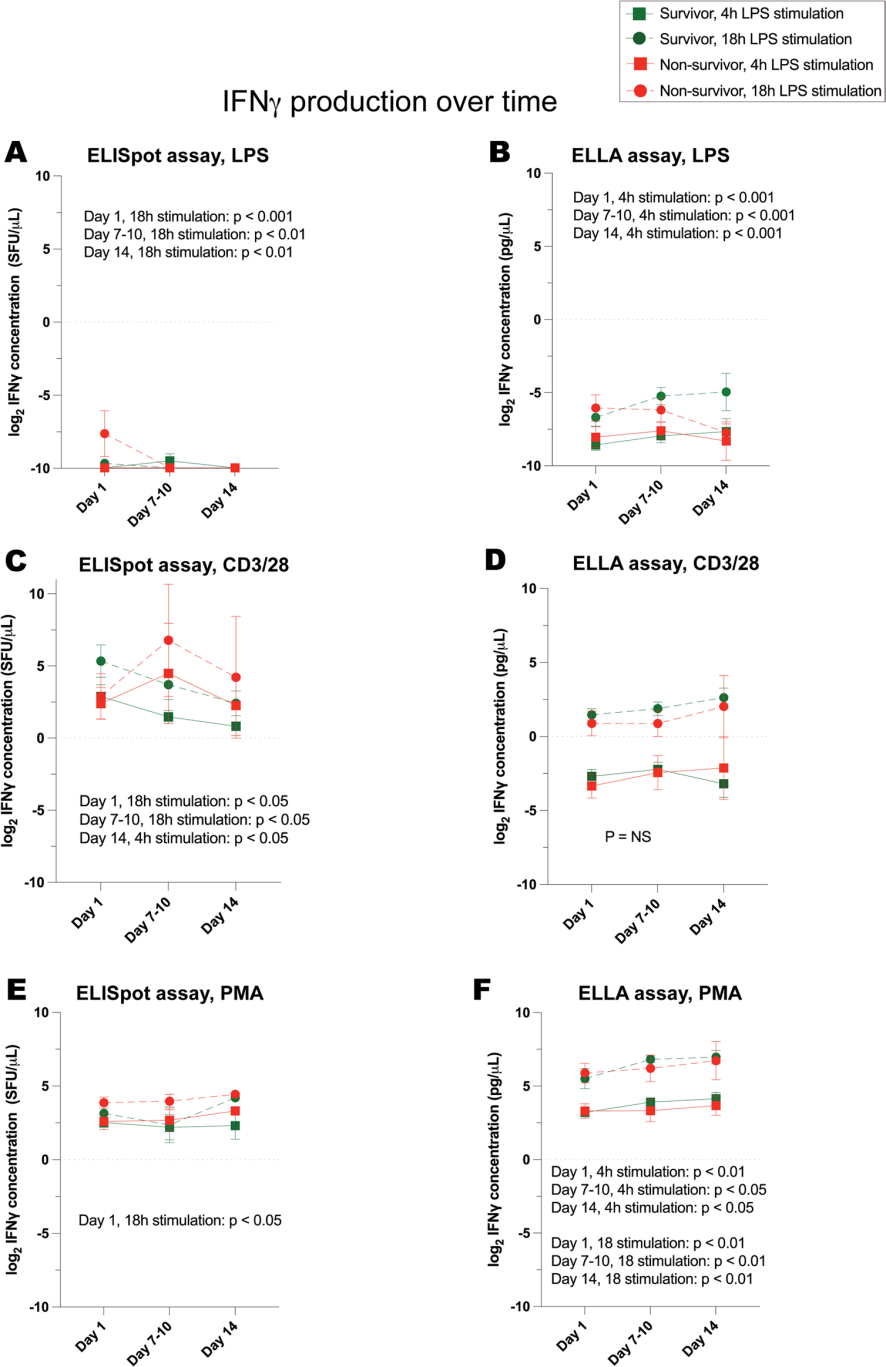
IFNγ production, over time, in response to stimulation of whole blood from critically ill survivors versus non-survivors. Cytokine concentrations are represented as log_2_-transformed values. **A** IFNγ concentration as measured by ELISpot and **B** ELLA immunoassays in response to LPS stimulation. **C** IFNγ concentration as measured by ELISpot and **D** ELLA in response to anti-CD3/anti-CD28 stimulation. **E** IFNγ concentration as measured by ELISpot and **F** ELLA in response to PMA stimulation. Day 1 denotes the day of study enrollment; dots represent mean values ± SEM. *P* values denote significant differences between stimulated TNF production in whole blood of survivors versus non-survivors, based on linear mixed-effects analysis. At day 1, *n* = 24 survivors, 8 non-survivors (7 critically ill and septic and 1 critically ill and non-septic)

**Fig. 9:**
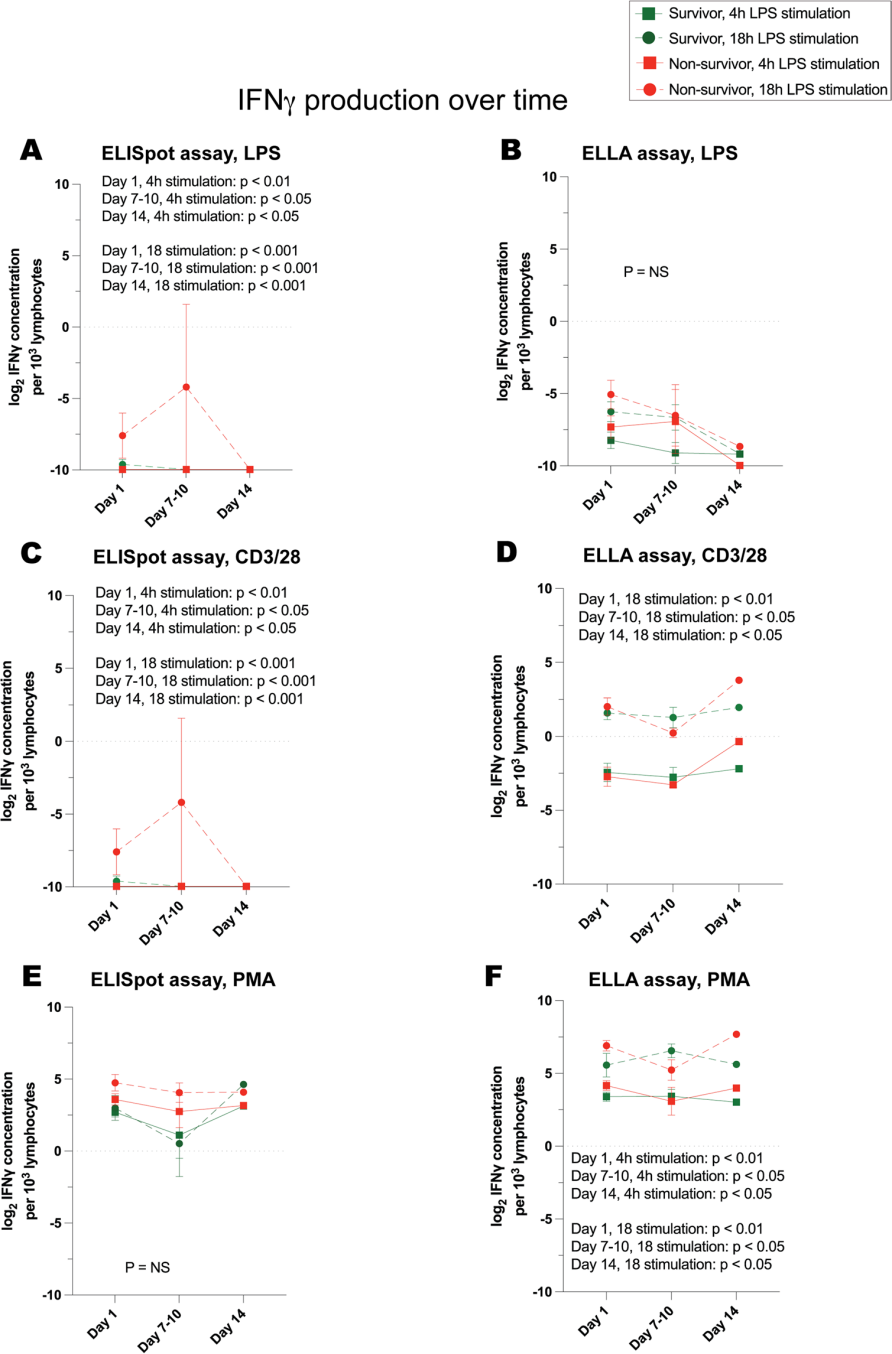
IFNγ production normalized to lymphocyte count, over time, in response to stimulation of whole blood from critically ill survivors versus non-survivors. Cytokine concentrations are represented as log_2_-transformed values. **A** IFNγ concentration as measured by ELISpot and **B** ELLA immunoassays in response to LPS stimulation. **C** IFNγ concentration as measured by ELISpot and **D** ELLA in response to anti-CD3/anti-CD28 stimulation. **E** IFNγ concentration as measured by ELISpot and **F** ELLA in response to PMA stimulation. Day 1 denotes the day of study enrollment; dots represent mean values ± SEM. *P* values denote significant differences between stimulated TNF production in whole blood of survivors versus non-survivors, based on linear mixed-effects analysis. At day 1, *n* = 24 survivors, 8 non-survivors (7 critically ill and septic and 1 critically ill and non-septic)

The association between patient survival and stimulated cytokine production on the first day of critical illness is illustrated in Fig. 5 (ELISpot cytokine measurements) and Fig. 6 (ELLA cytokine measurements). Subsequent patterns of cytokine production over the initial 14 days of critical illness are depicted in Figs. 7, 8, and 9. On the first day of illness, there was a notable correlation between ELISpot-measured TNF production (per 10^3^ monocytes) and patient survival (Fig. 5B). Specifically, non-survivors produced lower baseline concentrations of TNF at 18 h, lower concentrations of LPS-induced TNF at 18 h, and lower concentrations of TNF in response to 4 h/18 h anti-CD3/28 stimulation. This contrasted with ELLA-measured TNF and IFNγ production, where no significant differences were observed between survivors and non-survivors (Fig. 6).

### Impaired cytokine production persists over time in critically ill, septic, and non-septic patients

Using linear mixed-effects models, we examined the changes in stimulated cytokine production throughout the first 14 days of critical illness and assessed if these changes could predict survival (Figs. 7, 8, 9). We found a consistent association between patient mortality and TNF production measured by ELISpot after LPS stimulation (Fig. 7A). While a trend seemed to exist between decreased TNF production measured by ELLA and survival, it was not statistically significant based on the *P* values from our model (Fig. 7B).

IFNγ production in response to LPS, anti-CD3/anti-CD28, and PMA exhibited a more consistent association with survival over time. Notably, after 18 h of stimulation by LPS, anti-CD3/anti-CD28, and PMA, IFNγ concentrations were most indicative of survival when measured using the ELISpot immunoassay (Fig. 8). On the other hand, just 4 h of stimulation with LPS and PMA provided significant insight into survival trends when the ELLA immunoassay was employed for total, stimulated IFNγ quantification (Fig. 8). Once adjusted for lymphocyte counts, 4 h stimulation revealed associations between ELISpot-quantified IFNγ in response to LPS and anti-CD3/anti-CD28, and similarly, the relationship became clear for ELLA-quantified IFNγ post 4 h stimulation by anti-CD3/anti-CD28.

## Discussion

The present study describes a rapid assessment of immune cell function by ex vivo whole blood stimulation followed by one of two cytokine quantification methods: ELLA microfluidic-based, Simple Plex assay or the current, ‘gold standard’ ELISpot assay [11]. ELLA measurements were more precise, with a significantly lower coefficient of variation between replicate measurements. The dynamic range for ELLA-measured IFNγ and TNF measurements was also greater, detecting both low concentrations of TNF-induced IFNγ production and high concentrations of PMA-induced TNF and IFNγ production. The larger dynamic measurement range of the ELLA immunoassay also resulted in a lower incidence of measurements falling outside the reliable limits of detection. These may be considerable advantages when used in patients having highly heterogeneous immune responses. In addition, our findings indicate that TNF production following 4 h of LPS stimulation is comparable to that following 18 h of stimulation, both when ELLA and ELISpot assays are used for cytokine quantification. These findings may move us one step closer to developing a point-of-care assay of immune function in sepsis.

Prior investigations have evaluated different methods for measuring ex vivo cytokine production after whole blood stimulation and its impact on clinical outcomes in critically ill septic and non-septic patients. Endotoxin is the stimulant that is most commonly used for this purpose [22–27], although variations in study dose and duration of stimulation, as well as variable definitions of immune paralysis have made reports from individual investigations difficult to compare. Furthermore, while mortality may not be the optimal outcome for assessing the clinical impact of immune dysfunction, it may nonetheless be one of the most objective measures.

The research by Mazer et al. marked a significant progression towards creating a point-of-care-compatible immunoassay. Building upon this, our primary goal was to edge immune profiling closer to clinical application. Our study aimed to contrast the efficiency of the ELLA and ELISpot immunoassays in immune profiling [11]. The ELISpot assay has notable shortcomings: a lengthy 24-h turnaround time (comprising 18 h of ex vivo incubation of whole blood with immune stimulants and an additional 3–4 h of sample processing); susceptibility to optical artifacts; and a dependency on expert post-processing quality checks. On the other hand, our results show that the ELLA immunoassay effectively addresses several of these issues. Although no correlation was found between ELLA-measured TNF levels post-LPS stimulation and mortality, we did validate the prior report that TNF levels, after 18 h of LPS stimulation, correlate with patient mortality [11]. Notably, our study utilized only one fifth of the LPS dose employed by Mazer et al*.*, opting for 500 pg/ml LPS from *Salmonella* abortus equi, as opposed to their 2.5 ng/ml. This decision was informed by prior studies from Hall and colleagues [28–33]. Our results suggest a consistent link between LPS-induced TNF production and patient mortality.

High concordance between LPS-induced TNF concentrations at 4 h and 18 h suggests that 4 h of LPS stimulation may be adequate for assessing immune paralysis in sepsis. Furthermore, these results could be available to clinicians within 6 h of blood sampling (4 h of stimulant incubation followed by 90 min of sample processing by ELLA) and would require minimal expertise for sample processing. More importantly, time series data derived both by ELLA and ELISpot assays reveal important trends in TNF and IFNγ over the course of critical illness. While only ELISpot revealed a relationship between LPS-induced TNF production over time, both assays revealed decreases in total and lymphocyte-adjusted IFNγ concentrations over the first 2 weeks following sepsis/critical illness. This pattern is consistent with known activation of immune-inhibiting processes shortly following sepsis onset, designed to countermeasure the primary inflammatory response [34]. It is likely that both ELLA and ELISpot each provide valuable information, and that their combined use may provide a more comprehensive picture of immune function. The selection of ELLA versus ELISpot (or both) should thus depend on the resources available in the experimental setting, on the questions to be answered and on their potential therapeutic implications.

While the 25% mortality rate observed in our cohort is comparable to that reported in prior studies [35], the progressive decrease in cohort size resulting from patient deaths limits the conclusions that can be drawn from cytokine concentrations measured at days 7 and 14 of illness. Additional study limitations include the potential for inadvertent selection bias despite enrollment of consecutive patients, confounding by organ dysfunction, medications, and other clinical factors in critically ill, non-septic control group. External validity may further by limited by the study’s single-center design and by the disproportionate effect of any missing values on results derived from a small patient cohort.

In conclusion, functional alterations in cytokine production by myeloid and lymphoid cells can be detected within 6 h of blood collection using a microfluidic-based immunoassay system. The described method relies on rapid, sensitive, and automated cytokine detection coupled with complete blood count analysis. Sepsis-induced leukopenia needs to be factored into any immune evaluation, although automated complete blood analysis is now part of routine clinical care and is, therefore, unlikely to represent the rate-limiting step of functional immune phenotyping. The ELLA analyzer is compact and requires minimal user training for reliable sample processing, which are attractive features in rapid-paced clinical settings. While a larger clinical trial is needed to look at whether rapid immune profiling predicts clinical outcomes, our findings represent further progress towards the development of a clinical, point-of-care assay based on ex vivo stimulation of whole blood in sepsis.

### Supplementary Information

**Additional file 1: Figure S1.** Representative ELISpot images from healthy, critically ill/non-septic and critically ill/septic cohorts following lipopolysaccharide stimulation of whole blood for 4 h and 18 h. Each ELISpot well represents a membrane area of 0.26 cm2, exposed to a 50 ul of whole blood diluted ten-fold. (**A**) Healthy volunteers, (**B**) Two Critically ill, non-septic patients having survival of 60+ and 16-days, (C) Four critically ill and septic patients having survival of 60+, 16, 4 and 1-days. SFU = spot-forming units.** Figure S2:** Correlation between log2-transformed cytokine concentrations measured by ELISpot versus ELLA immunoassays. (**A**) Pooled IFNg measurements from all stimulant conditions in CINS, septic and healthy patients, (**B**) Pooled TNF measurements from all stimulant conditions in CINS, septic and healthy patients. CINS = critically ill and not septic patients.** Figure S3:** Spontaneous versus cytokine production following 4 h or 18 h of whole blood stimulation. (**A**) log2-transformed IFNg concentration measured following CD3/CD28 stimulation using ELISpot (top panels) or ELLA (bottom panels), (**B**) log2-transformed IFNg concentration measured following PMA stimulation using ELISpot (top panels) or ELLA (bottom panels), and (**C**) log2-transformed TNF concentration measured following LPS stimulation using ELISpot (top panels) or ELLA (bottom panels) immunoassays. ELISpot IFNy results for 18 h PMA stimulation are not shown since they were too numerous to count. n = 42 (22 sepsis, 10 critically ill and non-septic,10 septic patients). PMA/lono = PMA/ionomycin; LPS = lipopolysaccharide.

## Data Availability

The data sets used and/or analyzed during the current study are available from the corresponding author on reasonable request.
