# Supplementary material for: Comparison of whole blood cytokine immunoassays for rapid, functional immune phenotyping in critically ill patients with sepsis
Source: Intensive Care Med Exp. 2023 Oct 13;11:70. doi: 10.1186/s40635-023-00556-w (PMC10575832; doi:10.1186/s40635-023-00556-w)
Supplement: Supplementary file 1 — Additional file 1: Figure S1. Representative ELISpot images from healthy, critically ill/non-septic and critically ill/septic cohorts following lipopolysaccharide stimulation of whole blood for 4 h and 18 h. Each ELISpot well represents a membrane area of 0.26 cm2, exposed to a 50 ul of whole blood diluted ten-fold. (A) Healthy volunteers, (B) Two Critically ill, non-septic patients having survival of 60+ and 16-days, (C) Four critically ill and septic patients having survival of 60+, 16, 4 and 1-days. SFU = spot-forming units. Figure S2: Correlation between log2-transformed cytokine concentrations measured by ELISpot versus ELLA immunoassays. (A) Pooled IFNg measurements from all stimulant conditions in CINS, septic and healthy patients, (B) Pooled TNF measurements from all stimulant conditions in CINS, septic and healthy patients. CINS = critically ill and not septic patients. Figure S3: Spontaneous versus cytokine production following 4 h or 18 h of whole blood stimulation. (A) log2-transformed IFNg concentration measured following CD3/CD28 stimulation using ELISpot (top panels) or ELLA (bottom panels), (B) log2-transformed IFNg concentration measured following PMA stimulation using ELISpot (top panels) or ELLA (bottom panels), and (C) log2-transformed TNF concentration measured following LPS stimulation using ELISpot (top panels) or ELLA (bottom panels) immunoassays. ELISpot IFNy results for 18 h PMA stimulation are not shown since they were too numerous to count. n = 42 (22 sepsis, 10 critically ill and non-septic,10 septic patients). PMA/lono = PMA/ionomycin; LPS = lipopolysaccharide. [file 40635_2023_556_MOESM1_ESM.pdf]

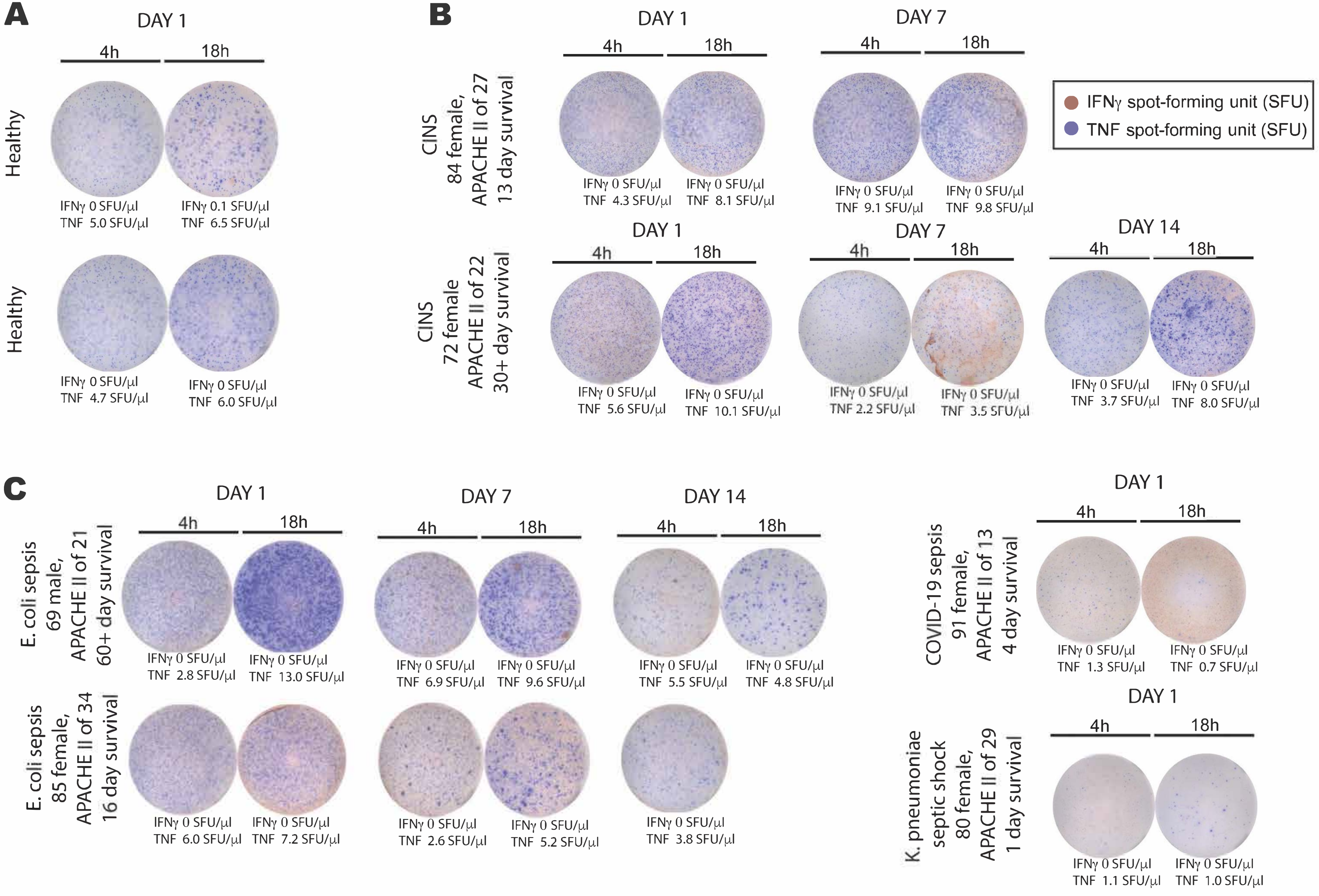


**Additional Figure S1:** Representative ELISpot images from healthy, critically ill/non-septic and critically ill/septic cohorts following lipopolysaccharide stimulation of whole blood for 4 h and 18 h. Each ELISpot well represents a membrane area of 0.26cm2, exposed to a 50 ul of whole blood diluted ten-fold. (A) Healthy volunteers, (B) Two Critically ill, non-septic patients having survival of 60+ and 16-days, (C) Four critically ill and septic patients having survival of 60+, 16, 4 and 1-days. SFU = spot-forming units.

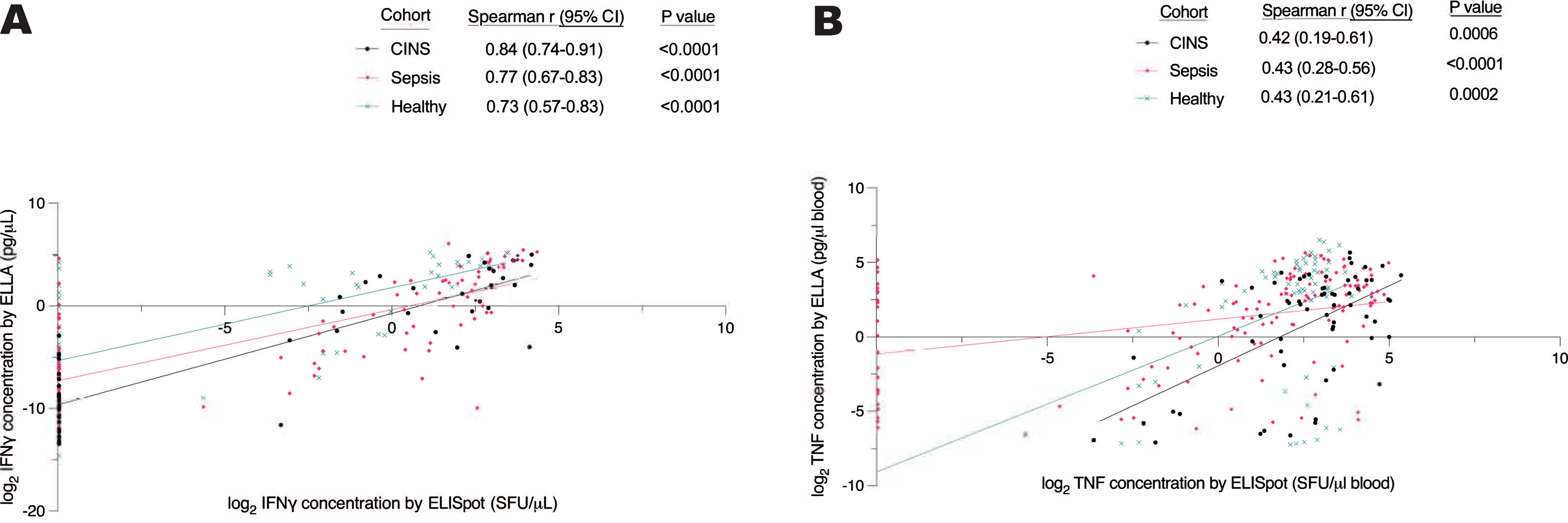


**Additional Figure S2:** Correlation between log₂-transformed cytokine concentrations measured by ELISpot versus ELLA immunoassays. (A) Pooled IFNγ measurements from all stimulant conditions in CINS, septic and healthy patients, (B) Pooled TNF measurements from all stimulant conditions in CINS, septic and healthy patients. CINS = critically ill and not septic patients.

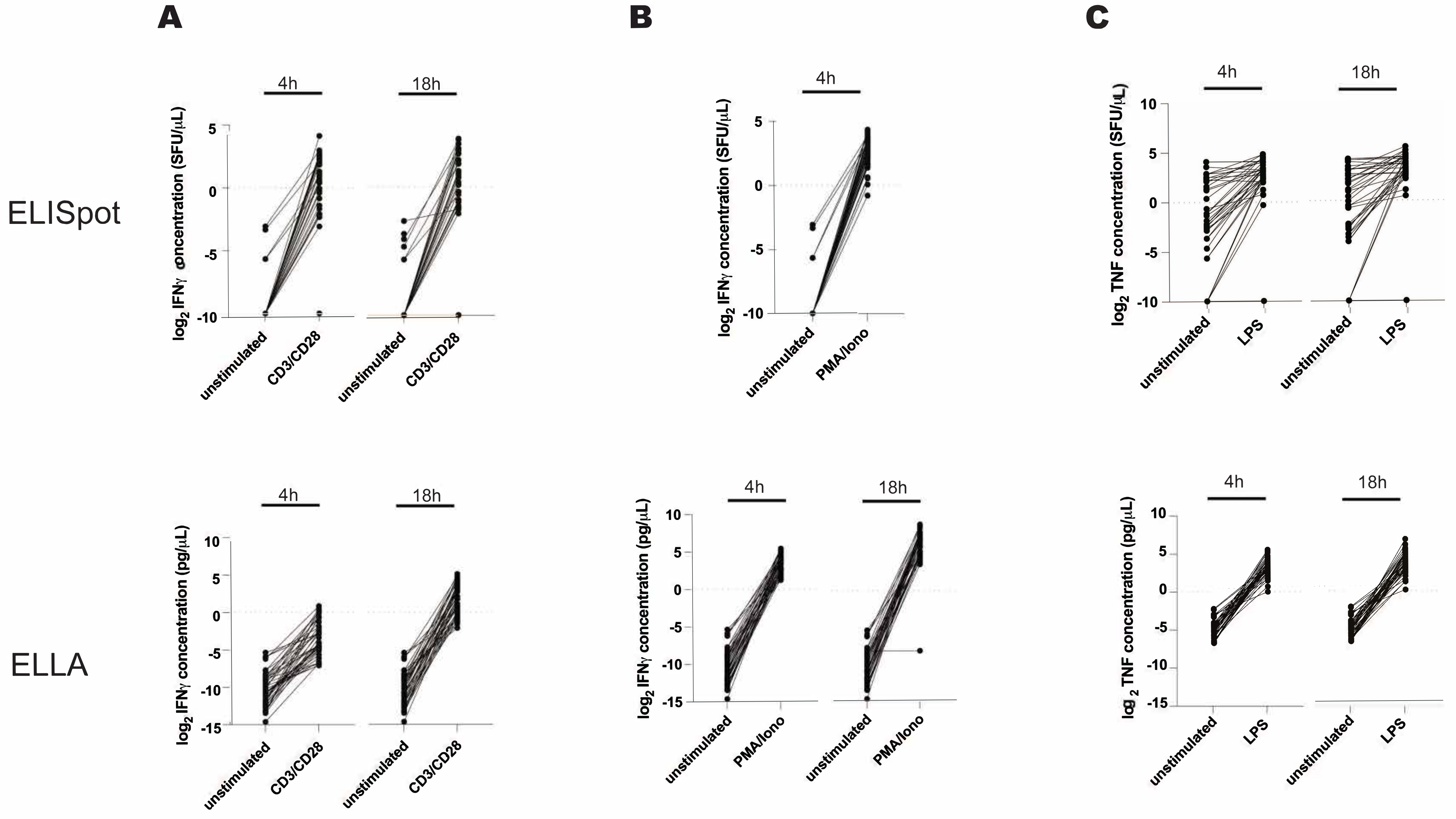


**Additional Figure S3**: Spontaneous versus cytokine production following 4 h or 18 h of whole blood stimulation. (A) $log_2$-transformed IFNγ concentration measured following CD3/CD28 stimulation using ELISpot (top panels) or ELLA (bottom panels), (B) $log_2$-transformed IFNγ concentration measured following PMA stimulation using ELISpot (top panels) or ELLA (bottom panels), and (C) $log_2$-transformed TNF concentration measured following LPS stimulation using ELISpot (top panels) or ELLA (bottom panels) immunoassays. ELISpot IFNγ results for 18 h PMA stimulation are not shown since they were too numerous to count. n=42 (22 sepsis, 10 critically ill and non-septic, 10 septic patients). PMA/Iono = PMA/ionomycin; LPS = lipopolysaccharide.
